# Endoscopic transfacet Decompression for Severe Lumbar Spinal Stenosis: A Technical Note, Illustrative Clinical Series, and Surgeon Survey Regarding Post-Decompression Instability

**DOI:** 10.3390/jpm15020053

**Published:** 2025-01-28

**Authors:** Kai-Uwe Lewandrowski, Álvaro Dowling, Choll Kim, Brian Kwon, John Ongulade, Kenyu Ito, Paulo Sergio Terxeira de Carvalho, Morgan P. Lorio

**Affiliations:** 1 Center for Advanced Spine Care of Southern Arizona, Division Personalized Pain Research and Education, Tucson, AZ 85712, USA; 2 Department of Orthopaedic Surgery, University of Arizona, Tucson Campus, Tucson, AZ 85712, USA; 3 Department of Orthopaedics, Fundación Universitaria Sanitas, Bogotá 110111, Colombia; 4 DWS Spine Clinic Center, CENTRO EL ALBA-Cam. El Alba 9500, Of. A402, Región Metropolitana, Las Condes 9550000, Chille; adowling@dws.cl; 5 Department of Orthopaedic Surgery, Faculdade de Medicina de Ribeirão Preto (FMRP), Universidade de São Paulo (USP), Ribeirão Preto 14040-900, Brazil; 6 Excel Spine Center, Minimally Invasive Center of Excellence, UCSD Medical Center, East Campus, 6719 Alvarado Road, Suite 304, San Diego, CA 92120, USA; ck11@chollkim.com; 7 New England Baptist Hospital, 125 Parker Hill Ave, Boston, MA 02120, USA; bkwon@nebh.org; 8 Department of Neurological Surgery, Washington University, 660 South Euclid, Campus Box 8057, St. Louis, MO 63110, USA; 9 Aichi Spine Institute, 41 Gohigashi, Takao, Fuso-cho, Niwa-gun, Aichi 480-0102, Japan; ken.it.yu@gmail.com; 10 Department of Neurosurgery, Pain and Spine Minimally Invasive Surgery Service, Gaffree Guinle University Hospital, Rio de Janeiro, 20270-004, Brazil; profdrpaulodecarvalho@gmail.com; 11 Advanced Orthopedics, 499 East Central Parkway, Altamonte Springs, FL 32701, USA; mloriomd@gmail.com

**Keywords:** lumbar spinal stenosis, endoscopic transfacet decompression, spine surgery, minimally invasive surgery, decompression, chronic pain, lumbar spine

## Abstract

**Background**: Lumbar spinal stenosis (LSS) remains a predominant cause of debilitating back and leg pain, affecting many aging populations. Traditional decompression surgeries can be invasive and pose significant risks and recovery time. This study elucidates the techniques and preliminary outcomes of endoscopic transfacet decompression in treating severe LSS. **Methods**: A retrospective review was performed on 65 patients with severe LSS who underwent endoscopic transfacet decompression. The patient outcomes were analyzed using the VAS for leg pain and the modified Macnab criteria. Pre-operative and post-operative scores were compared, and any complications were analyzed. An online survey was administered to 868 surgeons using Likert-scale ratings to evaluate surgeons’ experience with endoscopic decompression in patients with painful spondylolisthesis. The survey responses were analyzed using descriptive statistics and Polytomous Rasch analysis to evaluate surgeon endorsement. **Results**: The study included 65 patients, of which 29 (44.6%) were female and 36 (55.4%) were male, with a mean age of 65.79 ranging from 38 to 84 years. The available mean post-operative follow-up period was 31.44 months, ranging from 24 to 39 months. The VAS score for leg pain reduced significantly from pre-operative 7.54 ± 1.67 to 2.20 ± 1.45 by 5.34 ± 2.03 (*p* < 0.001) with a large effect size (Cohen’s d = 2.626). At the final follow-up, functional Macnab outcomes were reported as excellent by 20 (30.8%), good by 37 (56.9%), fair by 5 (7.7%), and poor by 3 (4.6%) of patients. There were no incidental durotomies, nerve root injuries, wound complications, or instances of post-operative instability. Only five patients (7.7%) developed post-operative dysesthesia. Incomplete decompression led to fair and poor outcomes in 8 (12.3%) patients. No revision surgeries were performed. post-operative instability was not observed. The surgeon survey corroborated these observations, where the polytomous Rasch analysis showed consensus on the effectiveness of the percutaneous endoscopic decompression of low-grade spondylolisthesis. Differential item functioning (DIF) analysis showed no significant bias in item responses between orthopaedic and neurosurgeons. **Conclusions**: The endoscopic transfacet decompression technique delineated herein showcased excellent Macnab outcomes in managing severe LSS, with a combined success rate of 87.7%. Patients also experienced a statistically significant reduction in leg pain. Dysesthesia rates were lower than with the transforaminal approach, likely because of limited exiting and traversing nerve root manipulation. This technique might represent a viable, less invasive alternative to open microsurgical dissection and decompression for patients with severe LSS, where fusion may be required. This approach was found to be highly accepted among endoscopic spine surgeons.

## 1. Introduction

Endoscopic lumbar stenosis decompression is commonly used to treat foraminal and lateral canal stenosis through the transforaminal approach. Severe narrowing of the central spinal canal may be challenging to treat with the endoscopic decompression platform, even in the most skilled hands [1]. One of the primary limitations of endoscopic lumbar stenosis decompression is its technical complexity [2,3]. The procedure requires a high level of skill and experience on the part of the surgeon due to the confined surgical area and the delicate nature of the small surgical field visualized on the video screen [2,4,5,6,7,8,9,10,11]. Using high-speed endoscopic power instruments and limited visualization of complex surgical anatomy through an endoscope may place additional demands on the endoscopic spine surgeon, thus increasing the learning curve. As a result, the complex endoscopic stenosis decompression of severe central canal stenosis may only be reserved for the most experienced surgeons proficient in endoscopic procedures [12].

The translaminar surgical corridor exploited by the full endoscopic interlaminar [13,14,15,16] and the uniportal bilateral endoscopic (UBE) decompression technique [17,18] emulate open laminotomy access to the lateral spinal canal by accessing and enlarging the interlaminar window by removing part of the rostral and caudal lamina and the medial portion of the facet joint complex comprised of the inferior and superior articular process [19,20]. While this surgical strategy is capable of better addressing the central canal stenosis by directly removing the compressive bony and soft tissue pathology consisting of hypertrophied ligamentum flavum and facet joint components than the transforaminal technique [21,22,23,24,25,26], it is also associated with a higher complication rate related to incidental durotomies [27,28,29,30] and epidural hematomas [31,32]. When these intra- and perioperative complications are encountered, there is not much recourse for the novice endoscopic spine surgeon, and conversion to open surgery is often the only bailout [33,34]. While the most skilled key opinion leaders have published endoscopic dural repair techniques [28,35,36,37,38], they require special training and additional equipment not part of the standard endoscopic tray setup. They are typically unavailable in the routine operating room setting. Considering recent publications indicating higher complication rates with the endoscopic translaminar decompression techniques [29,30,39,40], a new solution was required. Therefore, the authors of this article had a renewed interest in revisiting the safer transforaminal approach and how it could be modified to decompress the central canal more efficiently by going directly through the facet joint space.

The goal of the operation is a subtotal or total resection of the symptomatic facet joint complex by systematically removing the inferior articular process (IAP) from its attachments from the superior lamina and pars articularis and the superior articular process (SAP) from the inferior lamina and pedicle. The main objective of the transfacet approach is to overcome the shortcomings of the transforaminal and interlaminar and the other variations of translaminar endoscopic decompression techniques. In patients with severe central lumbar canal stenosis, the transforaminal approach may lead to incomplete decompression, and the interlaminar approaches may be associated with a higher incidence of incidental durotomies and bleeding. The authors stipulated that performing the majority of the decompression in the confined and safe compartment of the hypertrophied facet joint may improve clinical outcomes and reduce the risk of the endoscopic decompression procedure in patients with severe spinal stenosis. The key steps of the procedure consist of entering the facet joint space, egg-shelling out the majority of it before completing the foraminal and lateral and central canal decompression by removing the thinned-out bony remnants of the IAP and SAP. Therefore, this technique accesses the spine via the posterolateral approach by guiding the endoscopic working channel via a fluoroscopically placed guidewire directly into the joint space to begin the decompression.

## 2. Materials and Methods

### 2.1. Study Group

A cohort of 65 patients experiencing severe claudication symptoms and sciatica-type low-back and unilateral leg pain attributable to severe lumbar central canal and foraminal and lateral recess stenosis were included in this study. These patients underwent endoscopic transfacet decompression, with a follow-up period extending over two years post-operatively. Patient selection was stringent, adhering to selection criteria, including the failure of conservative management, radiologically confirmed lumbar stenosis consistent with the patient’s physical examination, and symptoms. The primary pain generator was identified preoperatively [41,42,43,44,45,46,47], employing peer-reviewed and published protocols. The spinal surgeons of this article performed the endoscopic transfacet decompression procedures on the more symptomatic side employing staged management protocols [42,44,48] for those few patients with bilateral symptoms (9/65), employing a standardized patient selection protocol utilized in this technical note article to minimize selection bias. Most patients (56/65) had unilateral symptoms.

### 2.2. Inclusion/Exclusion Criteria

The authors’ clinics implemented an endoscopic outpatient spinal surgery program to treat lumbar herniated discs and spinal stenosis. The transfacet endoscopic decompression procedure gains access to the spinal canal via the surgical lumbar facet joint space. It may improve clinical outcomes and reduce the risk of the endoscopic decompression procedure in patients with severe spinal stenosis. The patient inclusion criteria for this procedure are as follows:

1. Presence of clinical signs such as lumbar radiculopathy, dysesthesias, and decreased motor function.
2. Imaging evidence of severe central, foraminal, or lateral recess stenosis as shown in preoperative magnetic resonance images (MRI) and computed tomography (CT) scans defined as less than 100 mm^2^ on representative cross-axial sections.
3. Unsuccessful non-operative treatments, including physical therapy and transforaminal epidural steroid injections, for a minimum of 12 weeks.
4. Age between 35 and 85 years.

On the other hand, patients who are not suitable for the transfacet endoscopic lumbar based on the following exclusion criteria:

1. Segmental instability greater than Grade I spondylolisthesis or translational motion exceeding 8 mm on preoperative extension-flexion radiographs.
2. Infection.
3. Metastatic disease.

### 2.3. Endoscopic Technique

**Figure 1:**
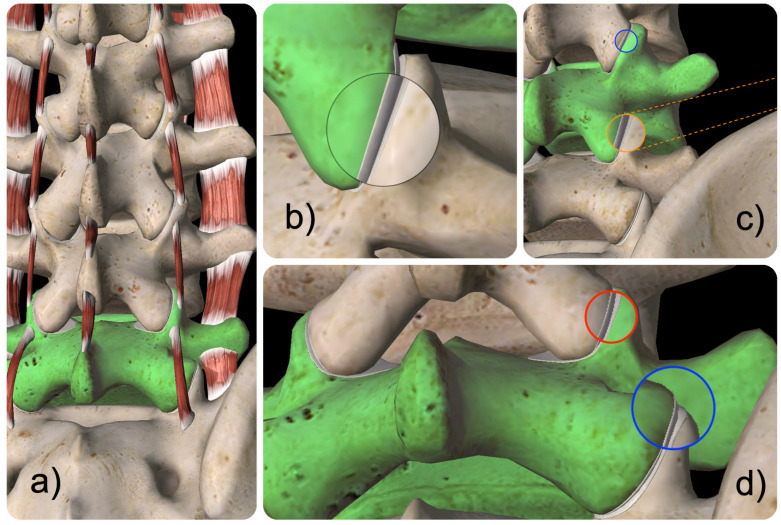
Model views of the posterior lumbar spine (**a**) showing the intertransverse muscles connecting the sacral alar to the transverse process of the L5 vertebral body (green) and the attachments of the intertransversari lumborum mediales muscles between one superior articular process to the next. A magnified view of the L4/5 facet joints is shown (**b**). Since the transfacet approach aims for the joint space, several capsular attachments and bony obstacles may be encountered along the surgical access corridor illustrated by the dashed orange lines to the exemplary approach to the L4/5 facet joint complex (**c**). Additional bony obstacles (**d**) may be encountered during the approach to the L4/5 (red lens) or L5/S1 (blue lens) facet joint pronounced by vertical collapse or other deformities typical of degenerative spine disease by the proximity of the L5 transverse process, hypertrophied facet joints, sacral alar, and posterior superior iliac spine.

**Figure 2:**
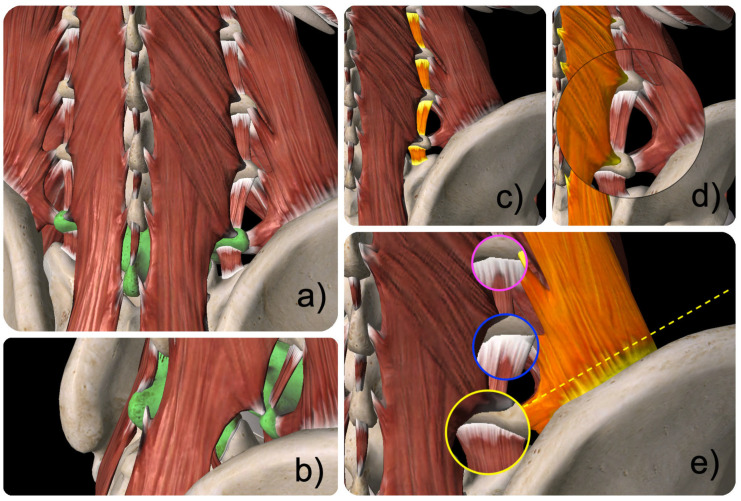
Schematic views of the muscular anatomy in the posterior lumbar spine (**a**) highlighting the L5 vertebral motion segment (green); (**b**) shows the muscular attachments of the multifidus, intertransverse lumborum medialis, and quadratus lumborum muscle at the lumbar facet joints; (**c**–**e**) illustrate the possible restrictions (highlighted in orange) to the lumbar facet joints through the intertransverse lumborum medialis (**c**), multifidus (**d**), and the quadratus lumborum muscle (**e**). The confluence of these three muscles at the L3/4 (pink lens), L4/5 (blue lens), and L5/S1 (yellow lens) facet joint complex is indicated. At the L5/S1 level, additional obstruction of the transfacet approach (dashed yellow line) may be encountered due to the tight aponeurotic attachments of the quadratus lumborum muscle.

The surgical approach in this procedure involves the endoscopic transfacet technique approaching the spine from a small skin incision placed between 5 to 7 cm posterolaterally from the midline at the surgical level. The relevant surgical anatomy is illustrated and reviewed in Figure 1 and Figure 2.

**Figure 3:**
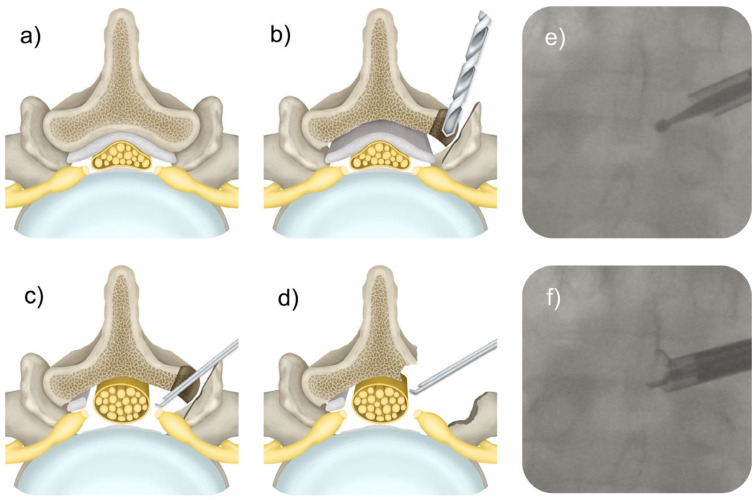
Shown are axial schematic views of the lumbar spine. Panel (**a**) illustrates central and lateral canal stenosis from facet hypertrophy. During the transfacet approach, the drill is placed into the facet joint (**b**) and further eggshelled out. An endoscopic Kerrison rongeur (**c**) is helpful during this portion of the procedure. At the end of the procedure, the paper-thin remnants of the superior (SAP) and inferior articular process (IAP) are then removed with a Kerrison rongeur at the base of the spinous process and lamina, thus accomplishing a complete removal of the facet joint complex from the pars rostrally to the pedicle caudally (**d**,**f**). Intraoperative fluoroscopy images show the intraarticular placement of the endoscopic drill bit (**e**). A rongeur can remove the remaining medial and lateral bony remnants of the SAP medially and the IAP laterally (**f**).

All surgeries are performed with patients in a prone position under general anesthesia, with the addition of local anesthesia using 0.25% bupivacaine. The targeted surgical facet joint is accessed under fluoroscopic guidance as follows:

1. Needle Placement: An 18 G (3 ½ inches in length) needle is carefully inserted into the lumbar facet joint complex at the surgical level to initiate the procedure. The targeting needle is ideally positioned in the lower part of the facet joint complex on the posterior–anterior (PA) and lateral view. The posterolateral targeting trajectory is best determined on the oblique view where the surgical facet joint space is best imaged. The needle tip should align with the medial interpedicular line close to the inferior pedicle on the PA view. Subsequently, the 18 G spinal needle’s trocar is removed, and a guide wire is introduced.
2. Placement of Working Cannula: A series of cannulated dilators with increasing diameters are deployed over the guide wire to gain access to the facet joint. Additionally, cannulated reamers measuring 7 and 9 mm in diameter or larger may be placed over the guidewire at the surgeon’s discretion to improve docking at the facet joint. The authors prefer to place a beveled working cannula first facing the lateral aspect of the facet joint to initiate the decompression at the SAP.
3. Intra-Facet Working Space: Once the working cannula is docked and the facet joint is videoendoscopically visualized, endoscopic power drills are employed to perform the initial foraminoplasty under direct visualization by creating a wide 8–10 mm working space within the facet joint, thereby slowly advancing the decompression into the foramen anteriorly. The authors’ preferred endoscope (asap Endosystems) is a standard foraminoscope with a 4.1 mm inner working channel and an outer 8.9 mm diameter working sleeve. Distortion of normal anatomy in hypertrophic facet joints is common, and one may find the ligamentum flavum when breaching the anterior portion of the facet joint rather than epidural fat or intervertebral disc. This critical step places the working cannula firmly into the joint space while minimizing the manipulation and, thus, the risk of dysesthesia of the exiting or traversing nerve root due to irritation of the dorsal root ganglia. It also establishes the depth of the necessary dissection to accomplish complete decompression and is an important landmark during the transfacet approach (Figure 3). A radiofrequency probe may also be handy to clean soft tissue attachments or to probe the extent of the decompression needed to alleviate neural structures in the foramen and the lateral recess from encroachment.
4. SAP Resection: After the initial foraminoplasty and establishment of the anatomical landmarks anteriorly, rostrally, and caudally, the authors’ preferred method is to decompress the lateral aspect of the facet joint complex by removing the SAP either in its entirety or as much as needed to decompress the exiting nerve root and visualize it in its entire course. Removing the SAP first has the advantage of freeing up the working cannula, which, to this point, is relatively tight in the facet joint. After the SAP is removed, the IAP can be more effectively decompressed with power burrs, drills, Kerrison rongeurs, and chisels because the surgeon can point the instruments medially, anteriorly, and posteriorly. After all, obstruction by the SAP is no longer problematic.
5. Partial Pediculolectomy: In vertically collapsed lumbar motion segments, an inferior partial pediculectomy and resection of the pars interarticularis may be necessary to achieve the desired lateral and central canal decompression.
6. Ring Apophysis Osteophytes: If required, the decompression of the traversing nerve root can be completed by drilling down the inferior ring apophysis and addressing any central disc bulge below the traversing nerve root and the central dural sac. In cases of a concurrent herniated disc, forceps and pituitary rongeurs are used to remove any extruded disc material. More often than not, the authors find contained herniations at this stage of the operation, which can be removed safely through a small annular window employing their hybridized outside-in/inside-out technique. For this step, the working cannular should be introduced into the disc space, which in geriatric patients is often hollow. Epidural bleeding can be controlled using a radiofrequency probe under saline irrigation.
7. Ligamentum Flavum Resection: The IAP resection exposes the ligamentum flavum, covering the central and lateral portions of the dural sac. Compared to the interlaminar approach, the transfacet approach facilitates the removal of the ligamentum flavum. It begins the decompression lateral to the ligamentum flavum rather than medial to it as dictated by the interlaminar window. Therefore, it is inherently safer as the remaining most medial portion of the IAP after having egg-shelled the decompression to the ligamentum flavum protects the neural elements throughout most of the bony decompression until it is removed during its final steps.
8. Over-the-top and Contralateral Decompression: Once the dural sac is decompressed and exposed on the approach side, the working cannula can be directed across the midline by undercutting the spinous process and removing bone and hypertrophied ligamentum flavum on the contralateral side. Alternatively, the exact transfacet decompression could be performed on the opposite side, creating a floating spinous process.

### 2.4. Surgeon Survey

The authors disseminated an online questionnaire through www.typeform.com to 793 potential surgeon participants using a link shared during the ISASS-sponsored Zoom webinar on 2 April 2024. Participants were requested to rate their confidence in obtaining favorable clinical outcomes with the endoscopic management Low-Grade Spondylolisthesis in the absence of post-operative instability. Ratings were given on a Likert scale ranging from 1 to 5, with 1 signifying Low and 5 High. This assessment was conducted at the beginning and end of the webinar to gauge changes in the participants’ levels of endorsement resulting from the lectures presented.

### 2.5. Statistical Analysis

Post-operative assessments were conducted at 3, 6, 12, and 24 months to evaluate pain (utilizing the Visual Analog Scale for leg pain), and functional outcomes were assessed via the modified Macnab criteria. Any complications, revision surgeries, or additional interventions were meticulously recorded. Comparative analyses of the pre-and post-operative VAS data were performed using descriptive and paired *t*-test statistical analyses calculating the means, mean difference, standard deviation, standard error, and effect size. The effect size was calculated as a quantitative measure that indicates the magnitude of the difference between two pre- and post-operative VAS scores. While the *p*-value from a *t*-test indicate whether there is a statistically significant difference, the effect size gives insight into how large or practical that difference is, helping to understand whether the difference is meaningful in a real-world context. The authors calculated Cohen’s d, which is calculated as the mean difference between the paired groups divided by the pooled standard deviation. Values for Cohen’s d are interpreted as “small effect size” (0.20 to 0.30), “medium effect size” (0.50), and “large effect size” (0.80 and above). The efficacy and safety of the procedure over the minimum of a two-year study period was analyzed using the Macnab data at the final follow-up. All statistical tests were conducted in SPSS Version 27.0 and Jamovi (version 2.3).

The chi-square test assessed the relationship between variables, while the Item Response Theory (IRT) module in Jamovi facilitated the Rasch analysis. A *p*-value of less than 0.05 was considered statistically significant, and a 95% confidence interval was applied to all statistical tests. The polytomous Rasch model, as detailed in the Part 1 report and outlined by Andrich, was utilized in this survey of surgeons. This model suggests that the characteristics of both the individual and the item determine the probability of a specific outcome in an empirical context. It models ordered response data by the likelihood of a response falling into categories such as “strongly agree”, “agree”, “disagree”, and “strongly disagree”. In the polytomous Rasch model, scoring x on an item indicates that an individual has surpassed x thresholds on a continuum while not surpassing the remaining m − x thresholds. Mathematically, the application of the Rasch model in this study is expressed as the log odds (or logit) of a person endorsing an item, reflecting the difference between the person’s ability or level of agreement and the item’s difficulty. The model uses chi-square fit statistics, outfit, and infit to evaluate the data’s fit to the model. The findings from the polytomous Rasch analysis are visually presented in the Wright Plot [49] and through Person Item Map Analysis [50].

The Rasch model is founded on equilibrium: for a reliable measure of individual traits, the number of items should be on par with the number of participants necessary for accurate item calibration. This balance is crucial in psychometrics to ensure the validity of the results obtained from the model. Azizan et al. suggest that administering an equal number of items and participants, for instance, 30 of each, under conditions of proper targeting and strong model fit, is likely to yield statistically robust measurements [51]. Specifically, the measures generated under these conditions are expected to maintain stability within ±1.0 logits at a 95% confidence interval. This equilibrium not only enhances the accuracy of the Rasch model but also solidifies its utility in reliably predicting responses on a standardized scale. Furthermore, the stability afforded by these parameters is vital for confirming the validity of the construct being studied and ensuring that observed data accurately represent the actual differences in the trait or ability being assessed rather than skewed by measurement inaccuracies or constraints related to the sample size.

## 3. Results

### 3.1. Clinical Series

**Figure 4:**
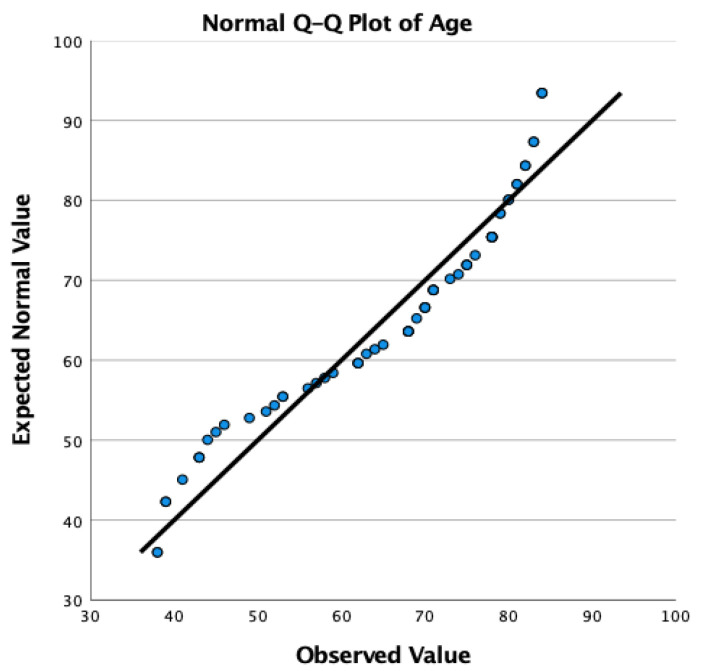
Quantile–Quantile (Q-Q) plot illustrating the age distribution of the 65 endoscopic transfacet decompression LSS patients. The Q-Q plot presents a graphical depiction of the age distribution among 65 patients. Each point on the plot represents a quantile of the patients’ age distribution against the corresponding quantile of a standard normal distribution. The x-axis denotes the expected quantiles of a normal distribution, while the y-axis represents the observed age quantiles of the sample. In the present plot, the close adherence of data points to the 45-degree reference line, suggests that the age distribution among our analyzed patients approximates a normal distribution, supporting the validity of subsequent parametric statistical analyses.

**Figure 5:**
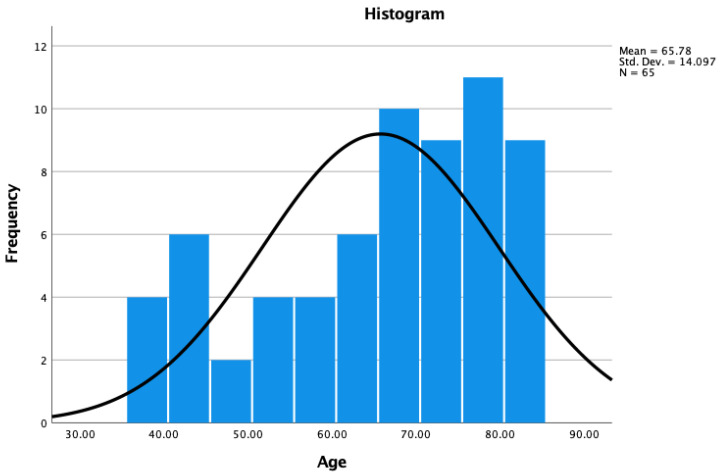
Histogram demonstrating the age distribution of 65 patients. The x-axis represents age bins, while the y-axis indicates the frequency of patients within each age bin. The data exhibits a bell-shaped curve, characteristic of a normal distribution, with the highest frequency observed in the age bin [38.00, 84.00]. The mean age of 65.7846 is represented at the peak of the distribution curve. The smooth curve overlaying the histogram is a Gaussian fit to the data, further emphasizing the normality of the age distribution within our patient cohort. This normal distribution allows for the application of parametric statistical tests in further analyses of the data.

The investigation encompassed 65 participants, consisting of 29 females (44.6%) and 36 males (55.4%), exhibiting a normal age distribution (Figure 4 and Figure 5) and an average age of 65.79, spanning from 38 to 84 years. A mean post-operative follow-up period of 31.44 months was achieved, with a span from 24 to 39 months (Table 1). Most patients (56/65) suffered from unilateral sciatica-type low-back pain and claudication leg symptoms due to severe central stenosis. Only 9 patients had bilateral symptoms. The more symptomatic side was surgically treated first in all 9 patients. Of these 9, 5 had a staged decompression on the lesser symptomatic contralateral side. The remaining four patients improved after the index transfacet decompression to a level where they did not deem surgical treatment of the contralateral side necessary.

At the concluding follow-up, 20 patients (30.8%) reported *excellent*, 37 (56.9%) indicated *good*, 5 (7.7%) declared *fair*, and 3 (4.6%) expressed *poor* functional Macnab outcomes. Incidental durotomies, nerve root injuries, wound complications, and post-operative instability were absent. post-operative dysesthesia occurred in 5 individuals (7.7%), and 8 (12.3%) experienced *fair* or *poor* outcomes due to incomplete decompression (Table 2).

Leg pain, evaluated using the VAS score, demonstrated a substantial reduction from a preoperative mean of 7.54 ± 1.67 to 2.20 ± 1.45, signifying a mean decrease of 5.34 ± 2.03 (*p* < 0.001; Table 3). The effect size measured as Cohen’s d was 2.626 and therefore the benefit from the lumbar endoscopic transfacet decompression for patients suffering from severe LSS was considered “large”. There were no instances of revision surgeries.

### 3.2. Case Example 1

Patient Profile: Age—80; Gender—Female; Presenting Chief Complaint—Severe lumbar spinal stenosis at L4/5 with facet hypertrophy.

Clinical Presentation: The patient was an 80-year-old female who presented with a history of chronic lower back pain and radiating pain down her left leg, particularly when walking or standing for prolonged periods. Neurological examination revealed weakness in dorsiflexion of the left foot, as well as hypoesthesia in the left L5 dermatome. The severity of her symptoms significantly impaired her mobility and quality of life.

**Figure 6:**
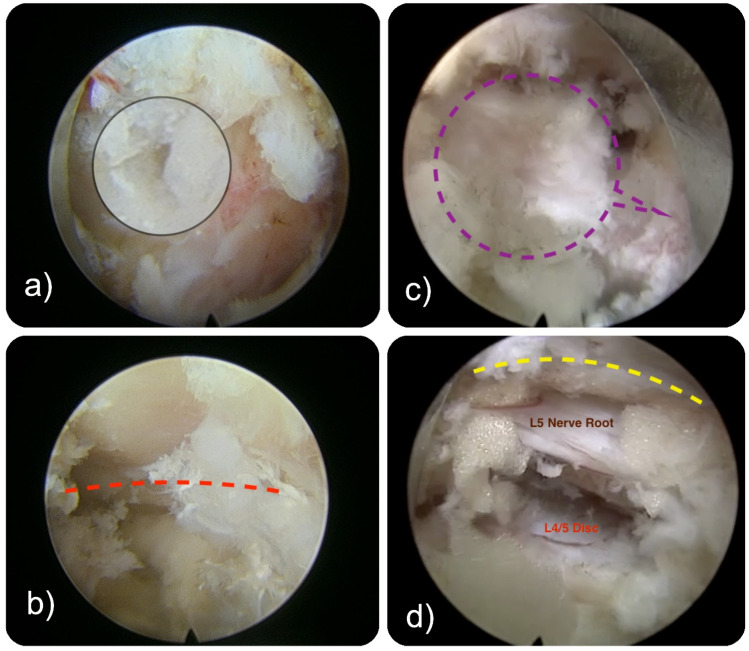
Intraoperative endoscopic views of the initial (**a**,**b**) and final (**c**,**d**) steps of the transfacet approach are shown. The black lens (**a**) offers the entry point into the lumbar facet joint at the tip of the superior articular process (SAP). Bony removal (**b**) with a power burr reveals the facet joint space identified with articular cartilage (dashed red line). The SAP (encircled by a purple line) is detached from the pedicle (purple callout arrow). The decompression is complete (**d**) when the lateral edge of the lamina is exposed (dashed yellow line). The L5 nerve root is decompressed in this exemplary view of the completed transfacet decompression after removing the entire facet joint complex, including the SAP and the inferior articular process (IAP). Typically, this decompression adequately decompresses the central canal on the access side.

Diagnostic Evaluation: Diagnostic imaging, including an MRI of the lumbar spine (Figure 6), demonstrated severe spinal stenosis at the L4/5 level with associated facet joint hypertrophy, predominantly affecting the left foraminal and lateral recess regions.

Intervention: Given the progressive nature of her symptoms and the anatomical findings on imaging, an endoscopic transfacet decompression at the L4/5 level was performed to address her foraminal, central, and lateral canal stenosis.

Outcome: post-operatively, the patient demonstrated a notable improvement in her lower back and leg pain, and exhibited enhanced left leg strength and sensory function. Physical therapy was initiated to facilitate recovery and promote optimal mobility. At follow-up appointments, she reported a marked improvement in her quality of life, being able to partake in daily activities with significantly reduced pain and improved function. The operative site healed without signs of infection or complication.

### 3.3. Case Example 2

Patient Profile: Age—61; Gender—Female; Presenting Chief Complaint—Symptomatic adjacent segment disease (ASD) at L4/5.

Clinical Presentation: The patient was a 62-year-old female, presented with persistent left-sided lower back pain and radicular pain radiating down her left leg, which had been progressively worsening over the past several months. The pain was notably exacerbated with extension of the lumbar spine and was consistent with her radiating neurological symptoms.

Diagnostic Evaluation: MRI imaging and diagnostic injections conclusively revealed symptomatic spine disease at the L4/5 level, demonstrating left-sided foraminal and central and lateral canal stenosis, likely a contributory factor to her present radicular symptoms and localized pain. In addition to the stenosis, there was evident facet joint hypertrophy and rigid Grade I spondylolisthesis at L4/5 on extension/flexion views.

**Figure 7:**
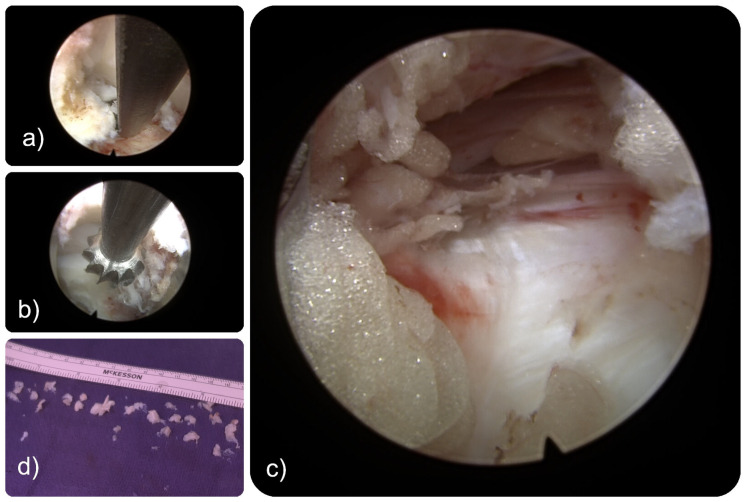
The intraoperative endoscopic views show the commonly employed decompression tools, including Kerrison rongeurs (**a**) and a non-sheathed low-spear, high-torque power drill (**b**), allowing for effective and rapid debris removal while facilitating continuous visualization without “white-out”. Complete central canal decompression can be achieved from the approach side, exposing the dural sac (**c**) and beyond over the top by removing large bony fragments (**d**).

Intervention: In consideration of her progressive symptoms and failed conservative care, the patient underwent a transfacet decompression at the L4/5 level on the symptomatic left side to remove bony and soft tissue stenosis from the foraminal area and the central and lateral canal (Figure 7).

Outcome: The patient’s recovery was uneventful, supplemented with a tailored physical therapy program aimed at improving strength, flexibility, and overall mobility. During subsequent follow-up appointments, she reported some transitory dysesthetic pain which was attributed to irritation of the dorsal root ganglion. It resolved within three weeks of supportive care measures including a transforaminal epidural steroid injection and oral gabapentin 300 mg tid for 2 weeks.

### 3.4. Case Example 3

Patient Profile: Age—73; Gender—Male; Presenting Chief Complaint—Severe lumbar central canal, foraminal, and lateral recess stenosis at L3/4.

Clinical Presentation: The patient is a 73-year-old male, presented with severe claudication symptoms and sciatica-type lower back and right leg pain, which severely impeded his mobility and overall quality of life. His claudication was evident by his inability to walk moderate distances and the requirement to frequently stop and assume a flexed-forward posture to alleviate his symptoms. The pain exhibited a radiating pattern down the leg and was associated with numbness and tingling, particularly when in an upright position.

Diagnostic Evaluation: An MRI scan of the lumbar spine disclosed severe central canal and bilateral foraminal and lateral recess stenosis at the L3/4 level, with accompanying nerve root compression. Electrodiagnostic studies also confirmed the presence of chronic right L3 and L4 radiculopathies. Trailed and failed conservative treatments included physical therapy and epidural steroid injections.

**Figure 8:**
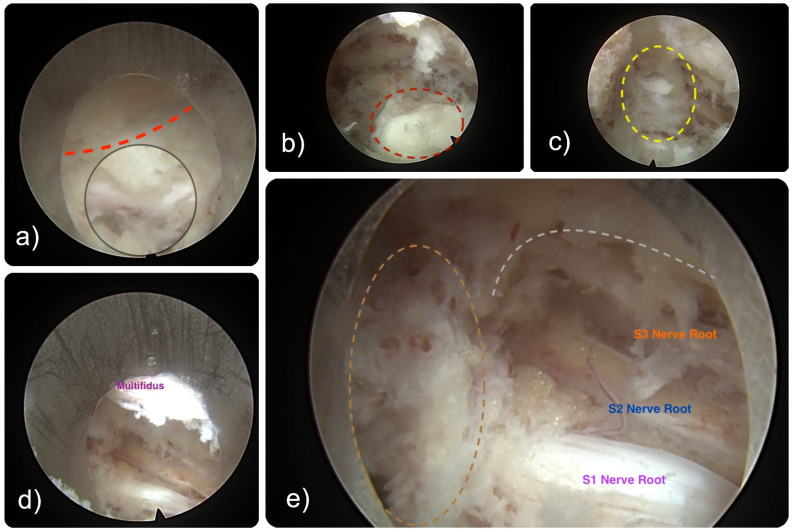
(**a**) Intraoperative endoscopic views of the L5/S1 transfacet approach to the central lumbar spinal canal. The joint space is shown (red dashed line). The dashed dark red circle illustrates the hypertrophied tip of the SAP (**b**) causing stenosis in the lateral canal. A sizeable bony fragment (yellow dashed circle) was pushed distally around the S1 pedicle while attempting to grab it with a pituitary rongeur (**c**). It was eventually retrieved (**d**,**e**) after mobilizing the fragment further. (**d**) The egg-shelling effect of the transfacet decompression technique is shown from a “bird’s-eye” view, showcasing the capsular attachment of the multifidus muscle and the large decompression site created. The thinned-out medial bony remnants of the SAP and IAP were removed with Kerrison rongeurs during the final steps of the decompression from the distal pedicle (brown dashed line) and the lateral edge of the lamina (grey dashed line), creating access to the central canal (**e**) and exposing the sacral nerve roots.

Intervention: Considering his age, clinical presentation, and failure to respond optimally to conservative management, a bilateral endoscopic transfacet decompression was performed. The nerve roots were decompressed bilaterally, relieving them from the impingement by removing the bony and ligamentous elements causing the stenosis (Figure 8).

Outcome: Post-operatively, the patient demonstrated a significant reduction in his claudication symptoms and sciatica-type pain, along with an improved ability to walk without requiring frequent rest periods. His post-surgical recovery was uncomplicated.

### 3.5. Surgeon Survey

The survey attracted an initial live online audience of 793 surgeons. Of these, 229 accessed the pre-webinar survey, with 154 starting it and 119 completing it, which translates to a 77.3% completion rate. The engagement level was consistent in the post-webinar phase: 298 surgeons accessed the survey, 169 started it, and 128 completed it, yielding a completion rate of 75.7%. Nearly three-quarters of respondents considered painful low-grade lumbar degenerative spondylolisthesis an appropriate indication for endoscopic decompression spine surgery with pre-webinar endorsement of 73.4% and 70.1% post-webinar, respectively. The post-webinar Person Item Map showed a significant endorsement shift, with an increase of agreement intensity for endoscopic decompression of lumbar spondylolisthesis being associated with favorable clinical outcomes with a shift of the mean logit locations to the right above the +1 logit, suggesting greater than 75% endorsement. Differential item functioning (DIF) analysis showed no significant bias in item responses between orthopaedic and neurosurgeons.

## 4. Discussion

Traditional transforaminal and interlaminar lumbar endoscopic approaches for spinal stenosis decompression described in a recent AO classification system [52], while providing a minimally invasive avenue and reduced post-operative morbidity, harbor intrinsic limitations that warrant attention. A quintessential drawback encompasses the steep learning curve [2,53,54], necessitating adept proficiency in endoscopic navigation [2], visualization [43,47,55], and instrument [26] manipulation to circumvent potential complications [56] and optimize outcomes. Particularly, comprehensive decompression of central stenosis or multifactorial stenosis involving both central and lateral recess or foraminal zones may be limited due to restricted visibility and access to specific anatomical structures, especially in excessive calcifications, hypertrophy, or osteophytes. Moreover, managing unforeseen intraoperative complications, such as dural tears [28,36], through the constrained endoscopic working channel poses tangible challenges and demands elevated surgical dexterity. The procedural efficacy in mitigating pain and restoring function in high-grade stenosis or spondylolisthesis cases is also a subject of ongoing research and debate. There is the potential for residual or recurrent stenosis, mainly when a substantial facet joint resection is needed. Careful preoperative planning and judicious patient selection are needed to avoid inadequate decompression to ensure optimal, enduring clinical benefit. Hence, while embodying a viable option for specific clinical scenarios, transforaminal and interlaminar lumbar endoscopic decompression for spinal stenosis mandates comprehensive understanding and sagacity in application to navigate its intrinsic shortcomings.

The authors proposed the transfacet technique as a safer yet effective way to remove large amounts of bony and soft tissue stenosis. Our data shows that the procedure is effective and reliably reduces pain and improves patient functioning, as corroborated by the favorable clinical outcomes in 87.7% of patients with excellent and good Macnab outcomes. The transfacet lumbar endoscopic decompression technique has several advantages, particularly in addressing lumbar central and lateral recess and foraminal stenosis, when juxtaposed against interlaminar or transforaminal endoscopic approaches. The transfacet approach is conducive to low-risk decompression in the spinal canal’s central and lateral aspects, thereby addressing multi-compartment stenosis with enhanced precision and efficacy. This mitigates the limitations observed in interlaminar and transforaminal approaches, where the surgeon may struggle with comprehensive decompression across all stenotic zones, especially when navigating rigid bony structures or significant osteophytic formations. transfacet endoscopic decompression is typically associated with low complication and dysesthesia rates. Our series observed no durotomy and only 7.7% of patients suffered from post-operative transitory dysesthesia from irritation of the surgical level’s dorsal root ganglion. Thus, this technique capitalizes on the hypertrophied facet joint being turned into a low-risk compartment from where complex lumbar stenosis involving multiple anatomical structures can be safely and effectively decompressed.

Post-operative instability was not observed in any patient, likely attributable to the inherent rigidity of the degenerative spine. This observation is corroborated by Haufe and Mork’s investigation into the effects of unilateral endoscopic facetectomy on spinal stability, concluded that such interventions did not compromise spinal integrity in their clinical series [57]. Their study underscores that careful removal of one facet joint, when performed endoscopically, does not significantly alter the biomechanical stability of the spine. Similarly, Youn et al. corroborated these findings with their clinical and radiological assessment of outcomes following endoscopic facetectomy for degenerative lumbar foraminal stenosis [58]. It further reinforces the argument against the development of instability. Their findings demonstrated not only significant clinical improvement in symptoms but also maintained spinal stability in the post-operative period, as evidenced by their radiological evaluations. Further, the Rasch analysis of survey data of 119 endoscopic spine surgeons, who routinely perform this operation, indicated high-intensity endorsement of endoscopic decompression of painful lumbar spondylolisthesis, suggesting that painful post-operative instability is not a problem they routinely run into.

The cited studies and presented survey data collectively argue that concerns regarding post-operative spinal instability following endoscopic unilateral transfacet decompression in the elderly, where significant degenerative changes with spontaneous sentinel fusions and increasing spinal rigidity due to vertical collapse are to be expected, are not substantiated when the procedure is performed by a skilled surgeon with the precision inherent to a target minimally invasive decompression procedure since much of the capsular and muscular attachment remain connected to the outer perimeter of the bony decompression crater. The cited and presented surgeon-experience-based and clinical evidence suggests that the endoscopic transfacet decompression, rather than precipitating instability, offers a targeted approach that alleviates symptoms of severe spinal stenosis without compromising the structural integrity of the spine to a level where it would become clinically relevant or prompt additional post-operative fusions due to unintended iatrogenic instability.

## 5. Conclusions

The lumbar endoscopic transfacet decompression may overcome some of the limitations of the transforaminal and interlaminar endoscopic lumbar stenosis decompression and offers numerous advantages over traditional open decompression surgeries due to its minimally invasive nature. In patients with severe central and lateral canal stenosis, the technical complexity may hinder achieving complete decompression with the other endoscopic techniques. Patients should be carefully selected and matched with the surgeon skill level, and their suitability for the operation should be carefully evaluated and discussed between patients and surgeons.

## Figures and Tables

**Table 1:** Patient demographic and post-operative follow-up data.

Demographics and Follow-Up	N	Minimum	Maximum	Mean
Age [Years]	65	38.00	84.00	65.7846
post-operative Follow-up [Months]	65	24.00	39.00	31.4462
Valid N (listwise)	65			
Gender	N	Percent	Valid Percent	Cumulative Percent
F	29	44.6	44.6	44.6
M	36	55.4	55.4	100.0
Total	65	100.0	100.0	

**Table 2:** Modified Macnab outcome criteria After transfacet decompression.

Modified Macnab Outcome	Frequency	Percent	Valid Percent	Cumulative Percent
Excellent	20	30.8	30.8	30.8
Good	37	56.9	56.9	87.7
Fair	5	7.7	7.7	95.4
Poor	3	4.6	4.6	100.0
Total	65	100.0	100.0	

**Table 3:** Paired sample tests, effect size, and confidence intervals.

Paired Samples Statistics			Mean	N		Std. Deviation		Std. Error Mean	
Pair 1	Preoperative VAS Score		7.5385	65		1.67777		0.20810	
	post-operative VAS Score		2.2000	65		1.44914		0.17974	
Paired Differences						t	df	Significance	
	Mean	Std. Deviation	Std. Error Mean	95% Confidence Interval of the Difference				One-Sided p	Two-Sided p
				Lower	Upper				
Preop VAS Score − Postop VAS Score	5.33846	2.03314	0.25218	4.83467	5.84225	21.169	64	<0.001	<0.001
Paired Samples Effect Sizes									
			Standardizer ^a^		Point Estimate		95% Confidence Interval		
							Lower	Upper	
Preop VAS Score − Postop VAS Score	Cohen’s d		2.03314		2.626		2.108	3.138	
	Hedges’ correction		2.04515		2.610		2.096	3.120	

^a^ The denominator used in estimating the effect sizes. Cohen’s d uses the sample standard deviation of the mean difference. Hedges’ correction uses the sample standard deviation of the mean difference, plus a correction factor.

## Data Availability

The individual clinical data is not made available due to privacy or ethical restrictions.
